# Long-term outcomes of stapled haemorrhoidopexy versus conventional haemorrhoidectomy: An updated systematic review, meta-analysis and trial-sequential analysis of randomized controlled trials

**DOI:** 10.1007/s00384-026-05080-3

**Published:** 2026-01-14

**Authors:** Sara Lauricella, Francesco Brucchi, Gianluca Mascianà, Giovan Battista Levi Sandri, Filippo Banchini, Gianlorenzo Dionigi, Diletta Cassini, Massimiliano Casati, Roberto Cirocchi

**Affiliations:** 1 Colorectal Surgery Division, Department of Surgery, Fondazione IRCCS Istituto Nazionale Dei Tumori, 20133 Milan, Italy; 2 Division of General Surgery, Istituto Di Ricovero E Cura a Carattere Scientifico (IRCCS) Istituto Auxologico Italiano, Milan, Italy; 3 Department of Pathophysiology and Transplantation, University of Milan, Via Festa del Perdono, 7, Milan, Italy; 4 Surgery Center, Colorectal Surgery Clinical and Research Unit, Fondazione Policlinico Universitario Campus Bio-Medico, University Campus Bio-Medico of Rome, 00128 Rome, Italy; 5 Digestive Surgery Unit, Fondazione Policlinico Universitario Agostino Gemelli IRCCS, Rome, Italy; 6 Department of General Surgery, Azienda USL of Piacenza, 29121 Piacenza, Italy; 7 Unit of General Surgery, Sesto San Giovanni Hospital, ASST Nord Milano, 20099 Sesto San Giovanni, Italy; 8 Department of General Surgery, Policlinico Di Monza, Monza, Italy; 9 Digestive and Emergency Surgery Unit, Santa Maria Hospital Trust, 05100 Terni, Italy

**Keywords:** Haemorrhoids, Haemorrhoidectomy, Stapled haemorrhoidopexy, PPH, Circular stapler, Milligan-Morgan, Ferguson, LigaSure, Harmonic scalpel

## Abstract

**Purpose:**

Stapled haemorrhoidopexy (SH) offers well-established short-term advantages over conventional haemorrhoidectomy (CH), but its long-term effectiveness remains controversial. This systematic review and meta-analysis aimed to compare long-term outcomes of SH versus CH in adult patients with grade II–IV haemorrhoids, incorporating Trial Sequential Analysis (TSA).

**Methods:**

MEDLINE, EMBASE and CENTRAL were searched from January 2001 to October 2025 for randomized controlled trials (RCTs) comparing SH and CH with a minimum follow-up of 12 months. Two reviewers independently performed study selection, data extraction and risk-of-bias assessment (RoB-2). Primary outcomes included recurrence, reintervention, anal function, and quality of life (QoL). Pooled risk ratios (RRs) with 95% confidence intervals (CIs) were calculated using random-effects models. TSA was applied to overall recurrence.

**Results:**

Seventeen RCTs including 1,041 SH and 1,031 CH patients were analysed. SH was associated with a higher risk of overall recurrence compared with CH (RR = 1.56,95%CI = 1.00–2.44;I^2^ = 48%). TSA showed that the accrued information size (1,913 patients) did not reach the required information size (2,608 patients), indicating that current evidence remains underpowered. Prolapse-related recurrence was significantly more frequent after SH (RR = 3.28,95%CI = 1.49–7.25;I^2^ = 12%), whereas bleeding-related recurrence did not differ between groups (RR = 1.20,95%CI = 0.63–2.26;I^2^ = 23%). No significant differences were found for reintervention, persistent anal pain, functional impairment or QoL.

**Conclusion:**

SH is associated with a higher risk of long-term recurrence, particularly prolapse-related recurrence, compared with CH, while long-term pain, functional outcomes and QoL appear broadly comparable. These findings suggest that, although SH may remain an option for selected patients, CH provides more durable anatomical correction, and procedural choice should balance early recovery against long-term durability.

**Supplementary Information:**

The online version contains supplementary material available at 10.1007/s00384-026-05080-3.

## Introduction

In contemporary practice, the management of symptomatic haemorrhoidal disease aims not only to achieve durable symptom control, but also to preserve anorectal function and improve patients’ quality of life (QoL) [1, 2]. The introduction of the transanal circular stapler by Longo in 1998 marked a major conceptual shift in the treatment paradigm [3]. Instead of excising haemorrhoidal tissue, stapled haemorrhoidopexy (SH) achieves a circumferential rectal mucosectomy with mucosal repositioning (anopexy), with the intention of restoring normal anatomy and reducing venous congestion and prolapse [4, 5].

Early comparative studies consistently demonstrated that SH provides several short-term advantages over conventional excisional haemorrhoidectomy (CH), including shorter operative time, less postoperative pain, reduced hospital stay, and faster return to normal activities [6]. These findings have been repeatedly confirmed by systematic reviews and meta-analyses over the past two decades [7, 8].

Over time, however, accumulating long-term evidence has reshaped this perspective. Recent data indicate that SH is associated with higher rates of recurrent prolapse, persistence of symptoms such as urgency or tenesmus, and an increased need for re-intervention compared with CH [9, 10]. The large pragmatic randomized eTHoS trial and its follow-up analyses have shown that, despite greater early morbidity, CH yields superior long-term QoL and more durable symptom resolution [11]. Moreover, recent reviews suggest that while both procedures remain safe, the long-term durability of SH appears limited, particularly in grade III–IV disease [7, 10].

These evolving insights support a patient-centred approach to procedural selection. SH continues to represent a valuable, minimally invasive option in carefully selected patients, whereas CH remains the most durable operation in terms of long-term anatomical and functional outcomes [7, 9–11].

Despite the widespread adoption of SH, important questions remain regarding its long-term effectiveness and impact on anorectal function. The last comprehensive meta-analysis specifically assessing long-term outcomes was published in 2009 [10]. Since then, multiple randomized controlled trials (RCTs) with extended follow-up have emerged [9, 11], yet no contemporary systematic review has focused exclusively on long-term results (≥ 12 months) using modern PRISMA methodology.

The present study aims to provide an updated, high-quality synthesis of RCT evidence comparing SH and CH in adult patients with grade II–IV haemorrhoids, focusing specifically on long-term outcomes (≥ 12 months). By analysing recurrence, re-intervention, anal pain, anal function, and QoL, this work will clarify whether the early advantages of SH persist over time and define its true long-term clinical impact.

## Methods

This systematic review and meta-analysis was conducted in accordance with the Preferred Reporting Items for Systematic Reviews and Meta-Analyses (PRISMA 2020) guidelines [12] and was prospectively registered on PROSPERO (International Prospective Register of Systematic Reviews; registration number CRD420251232921). Two reviewers (SL and FB) independently performed all phases of study selection, data extraction, and methodological quality assessment. Disagreements were resolved through discussion and, if necessary, by consultation with a third reviewer.

### Search strategy

A comprehensive literature search was performed in MEDLINE (via PubMed), EMBASE, and the Cochrane Central Register of Controlled Trials (CENTRAL) from January 2001 to October 2025, without language restrictions. The search combined free-text and MeSH terms related to both stapled and conventional techniques, using the following Boolean strategy: *(hemorrhoid* OR haemorrhoid* OR hemorrhoidectomy OR haemorrhoidectomy) AND (stapled OR stapler OR PPH OR stapled hemorrhoidopexy OR circular stapler OR Longo procedure OR anopexy) AND (conventional OR Milligan-Morgan OR Ferguson OR open hemorrhoidectomy OR closed hemorrhoidectomy OR diathermy OR electrocautery OR LigaSure OR Harmonic scalpel OR energy device).* Reference lists of relevant reviews and eligible randomized trials were also screened manually to identify any additional studies.

### Eligibility criteria

Only randomized controlled trials (RCTs) directly comparing stapled haemorrhoidopexy (SH) with conventional haemorrhoidectomy (CH) and reporting a minimum clinical follow-up of 12 months were included. Randomization had to involve prospective allocation to either SH or CH.

Conventional haemorrhoidectomy (CH) was defined as excisional surgery—open (Milligan–Morgan), closed (Ferguson), or energy-based techniques—entailing sharp or diathermic removal of haemorrhoidal tissue, anoderm, and perianal skin, with or without wound closure. Stapled haemorrhoidopexy (SH) was defined as circumferential resection of a rectal mucosal ring above the dentate line using a dedicated transanal circular stapler.

Trials were excluded if they: (i) lacked randomization or a direct comparison between SH and CH; (ii) reported only short-term outcomes (follow-up < 12 months); (iii) used modified stapling or non-circular devices not representative of standard SH; (iv) involved pediatric populations, emergency procedures, or concomitant major anorectal surgery; or (v) were non-comparative studies, conference abstracts without full text, animal studies, or duplicate publications (in which case the most complete dataset was retained).

### Study selection and data extraction

All titles and abstracts retrieved from the search were screened for relevance, and the full texts of potentially eligible studies were assessed in detail.

Data were extracted using a pre-standardized form capturing year of publication, country of origin, study design, sample size, patient demographics, haemorrhoid grade, details of the intervention and comparator, recurrence (prolapse, bleeding, or mixed), and any additional operations or other complications. Functional outcomes (urgency, continence disturbances, soiling, sensory symptoms, incomplete evacuation, and urinary dysfunction), and QoL measures (EQ-5D, EQ-VAS, SF-36, or validated Likert scales) were also collected, together with all other reported clinical endpoints.

Extracted data were cross-checked for accuracy before analysis.

### Quality assessment

Methodological quality and risk of bias were assessed independently by SL and FB using the Cochrane Risk of Bias 2 (RoB-2) tool [13]. (Five domains were evaluated: (i) the randomization process; (ii) deviations from intended interventions; (iii) missing outcome data; (iv) outcome measurement; and (v) selection of reported results. In accordance with Cochrane guidance, each randomized trial was classified as having *low risk of bias*, *some concerns*, or *high risk of bias*. (Suppl. Materials Table 1s). Any discrepancies were resolved through discussion.

The overall quality of evidence was judged by means of the GRADE (Grading of Recommendations, Assessment, Development and Evaluations) approach [14]. Based on the overall assessment the quality was divided into four grades (high, moderate, low or very low). The studies were either downgraded or upgraded in quality depending on whether the criteria of risk of bias, inconsistency, indirectness, imprecision, publication bias, large magnitude, dose response or effect of all plausible confounding factors were met. Authors FB and SL performed the GRADE assessment.

### Outcome measures

Long-term outcomes consistently focused on recurrence, reintervention, anal function, and QoL. Other reported complications (such as persistent pain at defecation, anal fissure, fistula, skin tags, or pruritus ani) were also recorded.

### Data synthesis and statistical analysis

Dichotomous outcomes were pooled using risk ratios (RRs) with corresponding 95% confidence intervals (CIs). Meta-analyses were performed with both fixed-effect (Mantel–Haenszel) and random-effects (DerSimonian–Laird) models; however, the random-effects model was prespecified as the primary analytical approach because of anticipated clinical and methodological heterogeneity across trials. When one or more cells contained zero events, a continuity correction of 0.5 was applied to all cells of the corresponding study. Trials with zero events in both treatment arms were excluded from effect estimation, as they provide no information on relative risk. Statistical heterogeneity was assessed using the I^2^ statistic and Cochran’s Q test. I^2^ values of 25%, 50%, and 75% were considered indicative of low, moderate, and high heterogeneity, respectively.

Predefined subgroup analyses were performed to assess recurrence according to its main clinical drivers (bleeding and prolapse). For each subgroup, pooled RRs were recalculated independently using the same random-effects framework. Sensitivity analyses using fixed-effect models were conducted to assess the robustness of the results but were not considered for primary inference.

Small-study effects and potential publication bias were explored visually using funnel plots centered on the fixed-effect pooled estimate.

For the primary outcome (overall recurrence), a Trial Sequential Analysis (TSA) was performed to evaluate the reliability of cumulative evidence while controlling for the risks of type I and type II errors inherent to sparse data and repeated significance testing. The required information size (RIS) and sequential monitoring boundaries were calculated under a two-sided testing framework with prespecified α and β error levels.

All analyses were performed using standard meta-analytic formulas implemented in Python. A p-value < 0.05 was considered statistically significant.

## Results

### Study selection

**Fig. 1:**
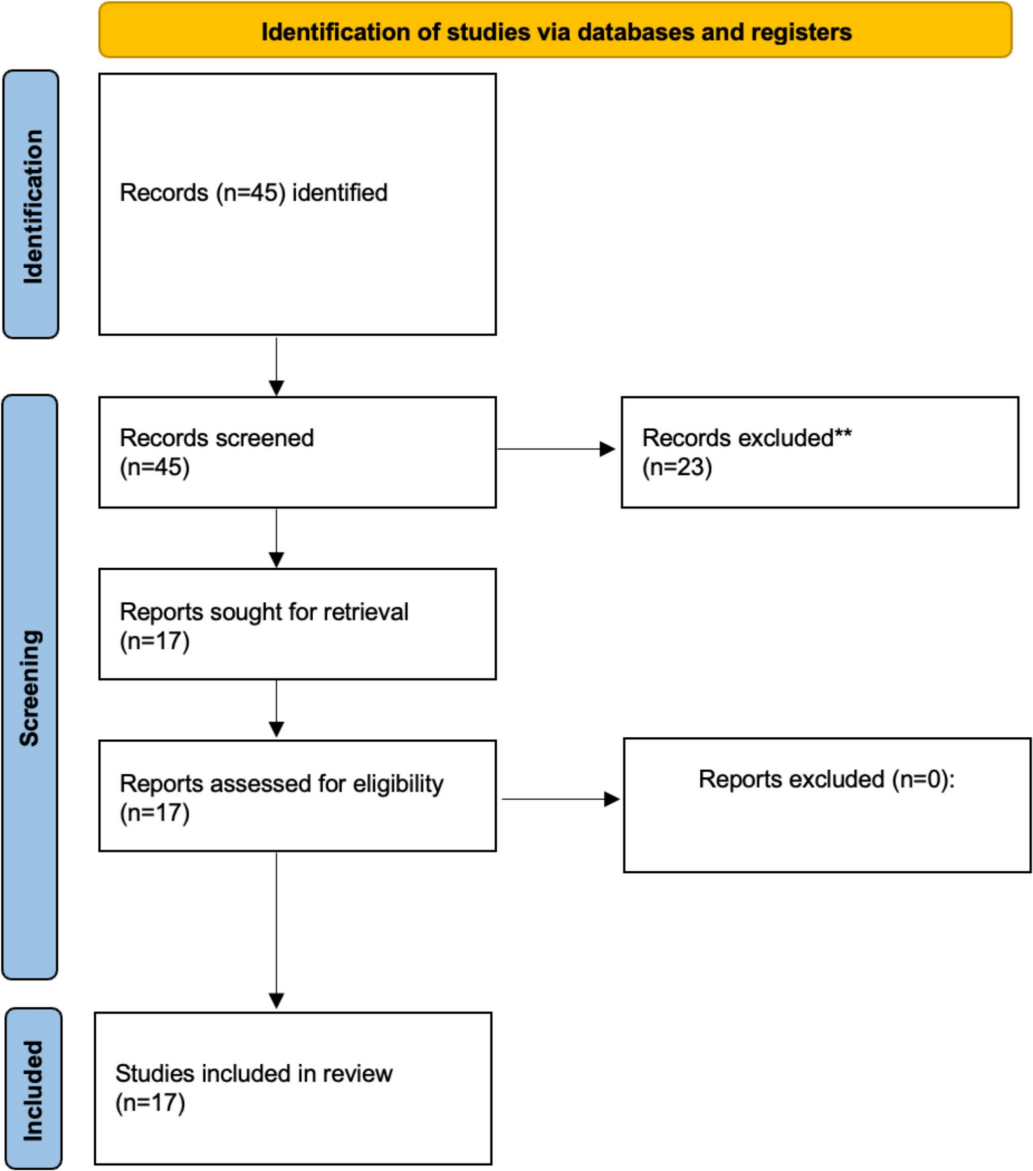
PRISMA flow-chart

The search strategy identified 45 records, of which 23 full-text articles were assessed for eligibility. After applying predefined inclusion criteria, 17 randomized controlled trials were included in the final quantitative synthesis. All studies compared SH with CH in adult patients with grade II–IV haemorrhoids and reported long-term outcomes (≥ 12 months) Fig. 1.

### Study characteristics

Seventeen randomized controlled trials published between 2001 and 2025 were included, comprising 1041 patients treated with SH and 1032 with CH. Baseline characteristics were comparable across studies.(Table 1)The mean age of participants was 48.2 years in the SH group and 48.1 years in the CH group. Sex distribution was available for 12 trials [9, 11, 15–24] and showed a comparable pattern between treatment arms: the SH group included 536 males (57%) and 406 females (43%), whereas the CH group included 456 males (55%) and 366 females (45%).The trials were conducted across Europe, North America and the Middle East. Stapled haemorrhoidopexy was performed using the standard circular stapling technique across all trials. Conventional haemorrhoidectomy showed greater variability, with 11 studies adopting the Milligan–Morgan procedure [11, 15–17, 20, 23–28], 3 using the Ferguson technique [9, 18, 21], and 2 employing an energy-based excisional approach [19, 22].

**Table 1:** Baseline characteristics of the included randomized controlled trials comparing stapled haemorrhoidopexy (SH) and conventional haemorrhoidectomy (CH)

Author	Country	SH (n)	Hemorrhoid grade – SH	M/F SH	CH (n)	Hemorrhoid grade – SH	M/F CH	Follow-up duration SH (months)	Follow-up duration CH (months)
Boccasanta et al. [16]	Italy	40	Grade IV (40)	15/25	40	Grade IV (40)	18/22	20 ± 7.8	20 ± 8.0
Shalaby and Desoky [20]	Egypt	100	II:13; III:32; IV:37; Prolapse:17	60/40	100	II:10; III:30; IV:40; Prolapse:20	64/36	12	12
Ortiz et al. [21]	Spain	27	III:17; IV:10	15/12	28	III:12; IV:16	17/11	15.2	15.9
Hetzer et al. [9]	Switzerland	20	II:6; III:14	15/5	20	II:6; III:14	14/6	12	12
Kairaluoma et al. [17]	Finland	30	Grade III (30)	13/17	30	Grade III (30)	19/11	12	12
Smyth et al. [28]	UK	20	Grade III (20)	NR	16	Grade III (16)	NR	37 ± 1.6	37 ± 1.6
Senagore et al. [18]	USA	77	Grade III (77)	49/26	79	Grade III (79)	58/19	12	12
Au-Yong et al. [29]	UK	11	Grade III (11)	NR	9	Grade III (9)	NR	42	42
Racalbuto et al. [23]	Italy	50	Grade III–IV (not separated)	31/19	50	Grade III–IV (not separated)	25/25	48	48
Ortiz et al. [22]	Spain	15	Grade IV (15)	8/7	16	Grade IV (16)	11/5	12	12
Van de Stadt et al. [24]	Belgium	20	Grades II–III (NR)	13/7	20	Grades II–III (NR)	13/7	46 (12–56)	46 (12–56)
Gravie´ et al. [27]	France	63	Grades III–IV (NR)	NR	63	Grade III–IV (NR)	NR	26.5 ± 3.1	26.5 ± 3.1
Ganio et al. [26]	Italy	43	Grades III–IV (NR)	NR	37	Grade III–IV (NR)	NR	87 (80–91)	87 (80–91)
Ammaturo et al. [25]	Italy	40	Grade III (40)	NR	40	Grade III (40)	NR	35 (24–51)	35 (24–51)
Kim Js et al. [15]	Germany	61	Grade III (61)	86/36	61	Grade III (61)	39/22	71.2 (34.0–128.2)	71.2 (34.0–128.2)
Watson et al. [11]	UK	389	II:86; III:243; IV:59	201/187	388	II:86; III:240; IV:60	197/189	24 (12.4–24.2)	24 (16.9–24.2)
Nada et al. [19]	Egypt	35	NR	30/5	35	NR	20/15	12	12

NR: not reported; Not separated: the information is reported in aggregated form without subgroup stratification.

Baseline haemorrhoid grade was broadly comparable across studies, with most trials enrolling predominantly Grade III–IV disease. Several RCTs included exclusively Grade III patients [15, 17, 18, 25, 28, 29], whereas Boccasanta et al. [16] and Ortiz et al. (2005) [22] recruited only Grade IV cases. Trials reporting full grade distribution demonstrated similar patterns between SH and CH—most notably Shalaby and Desoky [20] and the large eTHoS trial by Watson et al. [11] —where Grade III represented the most frequent presentation in both arms. Other studies enrolled Grade II–IV patients but did not provide subgroup counts [19, 23, 24, 26, 27]. Despite these reporting differences, baseline disease severity appeared well balanced between treatment groups.

Outcome assessment typically included validated symptom scores, anoscopy, anal manometry, and structured QoL questionnaires.

Long-term follow-up ranged from 12 to 87 months, providing the most comprehensive dataset currently available for assessing late outcomes of SH versus CH.

### Primary outcomes

#### Recurrence

Across the included trials, recurrence after haemorrhoid surgery was reported with substantial variability in definition and clinical presentation. Table 2. Overall, recurrence events were more frequently observed after SH, predominantly driven by prolapse-related recurrence, whereas bleeding-related recurrence appeared less consistently different between techniques. In the conventional haemorrhoidectomy group, prolapse recurrence was infrequent, and most recurrence events were related to postoperative bleeding rather than anatomical failure.

**Table 2:** Long-term recurrence outcomes in the included randomized controlled trials: prolapse-related and bleeding-related events

Author	Prolapse recurrence – SH (n)	Bleeding recurrence – SH (n)	Grade of SH patients affected	Prolapse recurrence – CH (n)	Bleeding recurrence – CH (n)	Grade of CH patients affected
Boccasanta et al. [16]	0	0	NR	0	2	NR
Shalaby and Desoky [20]	1	0	NR	2	0	NR
Ortiz et al. (2002) [21]	7	2	Grade III:2; Grade IV:5	0	1	NR
Hetzer et al. [9]	1	0	Grade II:1	1	0	Grade II:1
Kairaluoma et al. [17]	5	4	Grade II:5	0	1	NR
Smyth et al. [28]	0	5	NR	0	4	NR
Senagore et al. [18]	0	7	NR	0	4	NR
Au-Yong et al. [29]	0	0	NR	0	0	NR
Racalbuto et al. [23]	0	3	NR	0	8	NR
Ortiz et al. (2005) [22]	8	1	NR	0	1	NR
Van de Stadt et al. [24]	5	5	NR	0	6	NR
Gravie´ et al. [27]	4	1	NR	1	0	NR
Ganio et al. [26]	6	0	NR	4	0	NR
Ammaturo et al. [25]	5	7	NR	0	1	NR
Kim Js et al. [15]	NR*	NR	Grade IV:4	7 (not differentiated: prolapse/bleeding)	NR	Grade II:5 (only CH specified)
Watson et al. [11]	NR**	NR	NR	NR	NR	NR
Nada et al. [19]	0	1	NR	NR	NR	NR

NR: Not related.

*For Kim JS et al. [15], four recurrences in the SH group were not classified as prolapse- or bleeding-related; all occurred in patients with grade IV haemorrhoids.

**In the SH group, Watson et al. [11] reported an overall cumulative recurrence of 134 events among 317 patients (42%), without distinguishing between prolapse- and bleeding-related recurrence; in the CH group, 76 recurrences were reported among 300 patients (25%), also without distinction between prolapse-related and bleeding-related events.

##### Stapled Hemorrhoidopexy (SH)

Prolapse recurrence was documented in several trials. No cases were reported by Boccasanta et al. [16], Smyth et al. [28], Senagore et al. [18], Au-Yong et al. [29], Racalbuto et al. [23], or Nada et al. [19]. In contrast, Shalaby and Desoky [20] observed 1 recurrence. Ortiz et al. (2002) [21] documented 7 events, occurring in patients with grade III (n = 2) and grade IV (n = 5) haemorrhoids. Hetzer et al. [9] reported 1 recurrence in a patient with grade II disease. Kairaluoma et al. [17] documented 5 cases, all in patients who initially presented with grade II haemorrhoids. Ortiz et al. (2005) [22] reported 8 recurrences, Van de Stadt et al. [24] 5, Gravié et al. [27] 4, Ganio et al. [26] 6, and Ammaturo et al. [25] 5 cases. Kim et al. [15] reported four cases of recurrence, without specifying whether these were due to prolapse or bleeding; all occurred in patients with grade IV haemorrhoids.

Recurrent bleeding was less consistently reported. No bleeding-related recurrences, or no specific data, were provided by Boccasanta et al. [16], Shalaby and Desoky et al. [20], Hetzer et al. [9], Ganio et al. [26], Au-Yong et al. [29], or Watson et al. [11] Ortiz et al. (2002) [21] identified 2 cases of recurrent bleeding, Smyth et al. [28] 5, Senagore et al. [18] 7, Racalbuto et al. [23] 3, Ortiz et al. (2005) [22] 1, Van de Stadt et al. [24] 5, Gravié et al. [27] 1, Kairaluoma et al. [17] 4, and Ammaturo et al. [25] 7 cases. Nada et al. [19] described 1 bleeding-related recurrence, although haemorrhoid grade was not reported.

Only Watson et al. [11] provided an overall cumulative recurrence rate, reporting 134 events among 317 patients (42%), without distinguishing between prolapse-related and bleeding-related events.

##### Conventional Hemorrhoidectomy (CH)

Prolapse recurrence was infrequent in the CH group. No cases were reported by Boccasanta et al. [16], Ortiz et al. (2002) [21], Kairaluoma et al. [17], Smyth et al. [28], Senagore et al. [18], Au-Yong et al. [29], Racalbuto et al. [23], Ortiz et al. (2005) [22], Van de Stadt et al. [24], and Ammaturo et al. [25] Shalaby and Desoky [20] reported 2 recurrences, Hetzer et al. [9] described 1 case in a patient with grade II disease, and Gravié et al. [27] and Ganio et al. [26] reported 1 and 4 cases, respectively. Kim et al. [15] documented 7 recurrences overall but did not differentiate between prolapse and bleeding. Nada et al. [19] reported 7 recurrences overall, without specifying whether these were due to prolapse or bleeding.

Recurrent bleeding was variably documented. Boccasanta et al. [16] reported 2 cases, Ortiz et al. (2002) [21] 1, Kairaluoma et al. [17] 1, Smyth et al. [28] 4, Senagore et al. [18] 4, Racalbuto et al. [23] 8, Ortiz et al. (2005) [22] 1, Van de Stadt et al. [24] 6, and Ammaturo et al. [25] 1 case. No bleeding recurrences were reported by Shalaby and Desoky [20], Hetzer et al. [9], Gravié et al. [27], or Ganio et al. [26]. None of the studies reporting bleeding recurrence specified the haemorrhoid grade associated with the event.

Watson et al. [11] provided an overall cumulative recurrence rate of 76 events among 300 patients (25%) in the CH arm, again without distinguishing between prolapse and bleeding.

#### Meta-analysis (Fig. 2)

**Fig. 2:**
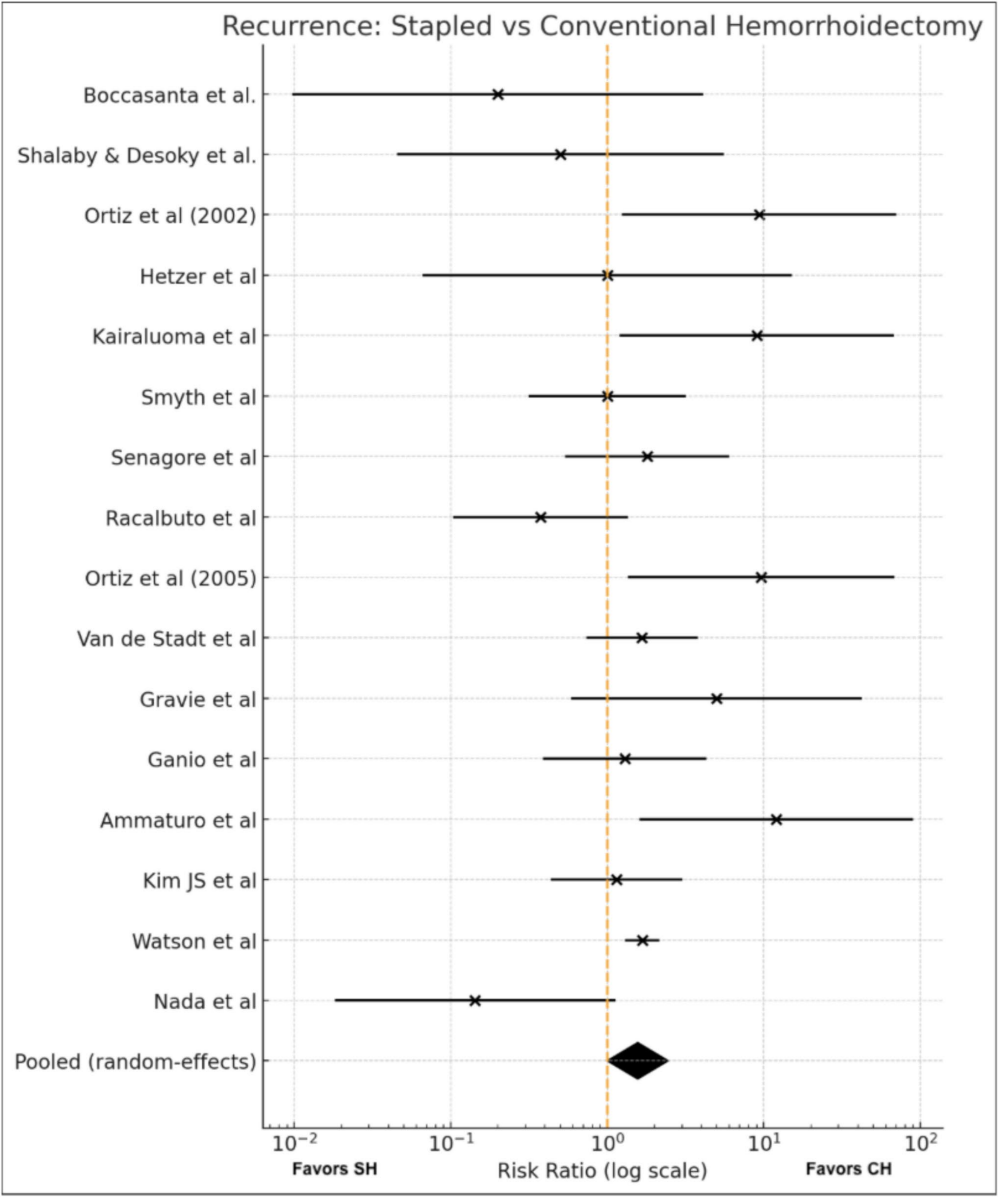
Overall recurrences

Seventeen trials [9, 11, 15–29] reported postoperative recurrence, including a total of 2,073 patients. One study [29] with zero events in both arms was excluded from the quantitative synthesis. Using a random-effects model, SH was associated with a statically significant increased risk of recurrence compared with the CH (RR 1.56, 95% CI 1.00–2.44; p = 0.05). Statistical heterogeneity was moderate (I^2^ = 48%). As a sensitivity analysis, recurrence rates were re-analyzed including only studies with a follow-up longer than 12 months, which did not show a statistically significant difference between SH and CH (Suppl. Materials).

#### Trial Sequential Analysis (TSA)

**Fig. 3:**
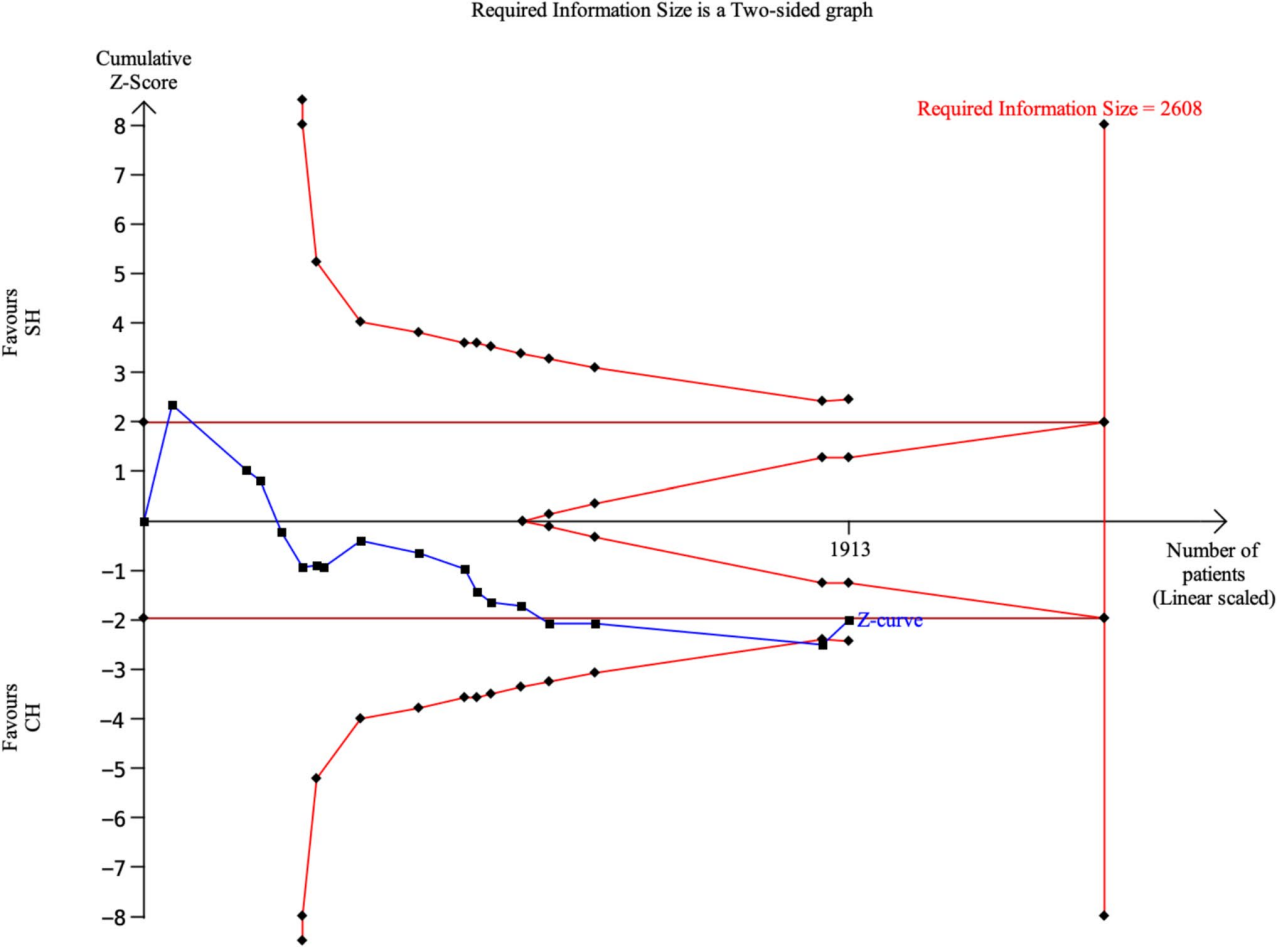
Trial sequential analysis—overall recurrences

A trial sequential analysis was performed to assess whether the accumulated evidence was sufficiently robust to support a definitive conclusion while controlling for random errors due to sparse data and repeated significance testing (Fig. 3). The cumulative Z-curve did not cross the monitoring boundaries for benefit or harm at any point during the accumulation of evidence.

The accrued information size (1,913 patients) did not reach the required information size (RIS) of 2,608 patients, calculated according to the anticipated effect size, event proportion, and the predefined type I and type II error risks. Thus, the TSA indicates that the currently available evidence remains underpowered and insufficient to confirm or refute a clinically meaningful difference in recurrence between SH and CH.

#### Recurrence (bleeding) Meta-analysis (Fig. 4)

**Fig. 4:**
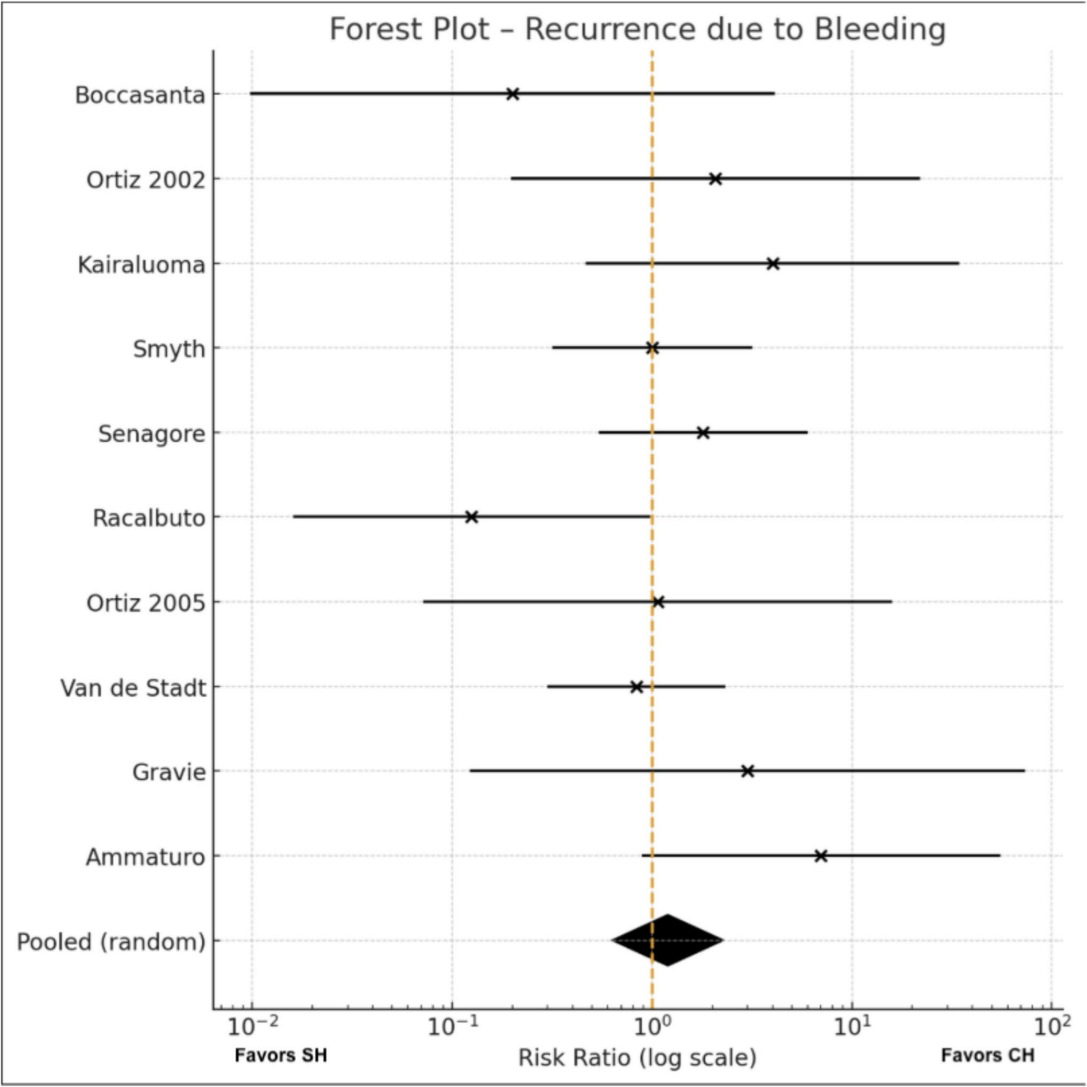
Bleeding recurrences

Ten trials [16–18, 21–25, 27, 28] reported bleeding-related recurrence and were included in this subgroup analysis. Event rates varied across studies, with some reporting very few recurrences in either arm. Using a random-effects model, no significant difference was observed between stapled and conventional haemorrhoidectomy in the risk of recurrence due to bleeding (RR 1.20, 95% CI 0.63–2.26; p = 0.58). Heterogeneity was low to moderate (I^2^ = 23%), indicating acceptable consistency across the included trials.

Visual inspection of the funnel plot did not reveal clear evidence of small-study effects or publication bias, although interpretation is limited by the small number of studies; the full plot is provided in the Supplementary Materials Fig. 2s.

#### Recurrence (prolapse) Meta-analysis (Fig. 5)

**Fig. 5:**
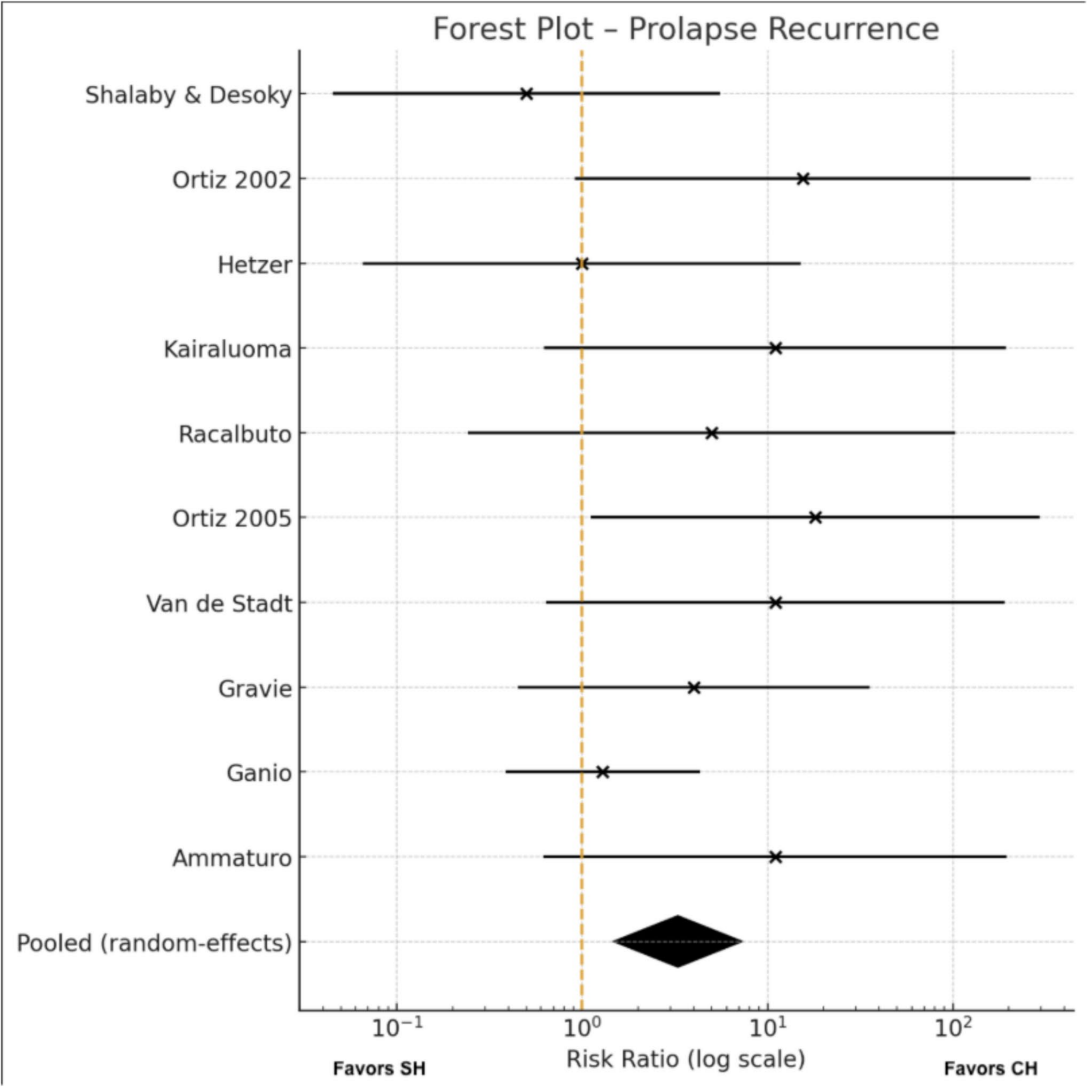
Prolapse recurrences

Ten trials [9, 17, 20–27] reported recurrence related to mucosal prolapse and were included in this subgroup analysis. Across these studies, events occurred more frequently after SH, with several trials reporting zero prolapse recurrences in the conventional arm.

Using a random-effects model, SH was associated with a significantly higher risk of prolapse recurrence compared with the conventional technique (RR 3.28, 95% CI 1.49–7.25; p = 0.003). Statistical heterogeneity was low (I^2^ = 12%), indicating good consistency among study estimates and supporting the robustness of this finding.

Inspection of the funnel plot suggested some asymmetry, although interpretation is inherently limited by the small number of studies; the full plot is provided in the Supplementary Materials Fig. 3s.

#### Additional operations

Additional operations across the included studies were infrequent overall, although reporting was often heterogeneous and incomplete. Outcomes differed SH and CH, with only a minority of trials providing detailed information regarding the type and indication of reintervention.

##### Stapled Hemorrhoidopexy (SH)

Most studies reported no additional surgical procedures following SH [9, 16, 17, 19–23, 28, 29].

Among trials documenting reinterventions, Senagore et al. [18] reported two procedures, both consisting of rubber band ligation. Van de Stadt et al. [24] described four reinterventions, all performed as redo surgery for recurrent prolapse and/or external swelling. Ganio et al. [26] also reported two additional procedures, again rubber band ligations. Ammaturo et al. [25] described five reinterventions, including three repeat SH procedures and two conventional haemorrhoidectomies. Kim et al. [15] documented one additional operation (excision of skin tags). Watson et al. [11] reported the highest number of reinterventions (n = 34), although the specific procedures were not specified. Gravié et al. [27] noted the presence of additional operations but did not provide further details.

##### Conventional Hemorrhoidectomy (CH)

Additional operations were likewise infrequent in the CH group. No reinterventions were documented in thirteen trials [9, 16, 17, 19–25, 27–29].

Among studies reporting additional procedures, Senagore et al. [18] described eleven reinterventions, including fistulotomy (n = 2), anal sphincterotomy (n = 1), excision of perianal skin tags (n = 1), and examination under anesthesia (n = 1). Ganio et al. [26] reported one additional operation (rubber band ligation). Kim et al. [15] documented seventeen reinterventions, including excision of skin tags (n = 10), anal polyp excision (n = 5), and cryptoglandular fistula excision (n = 2). Watson et al. [11] reported twenty-three additional operations, although the specific procedures were not detailed.

#### Meta-analysis (Fig. 6)

**Fig. 6:**
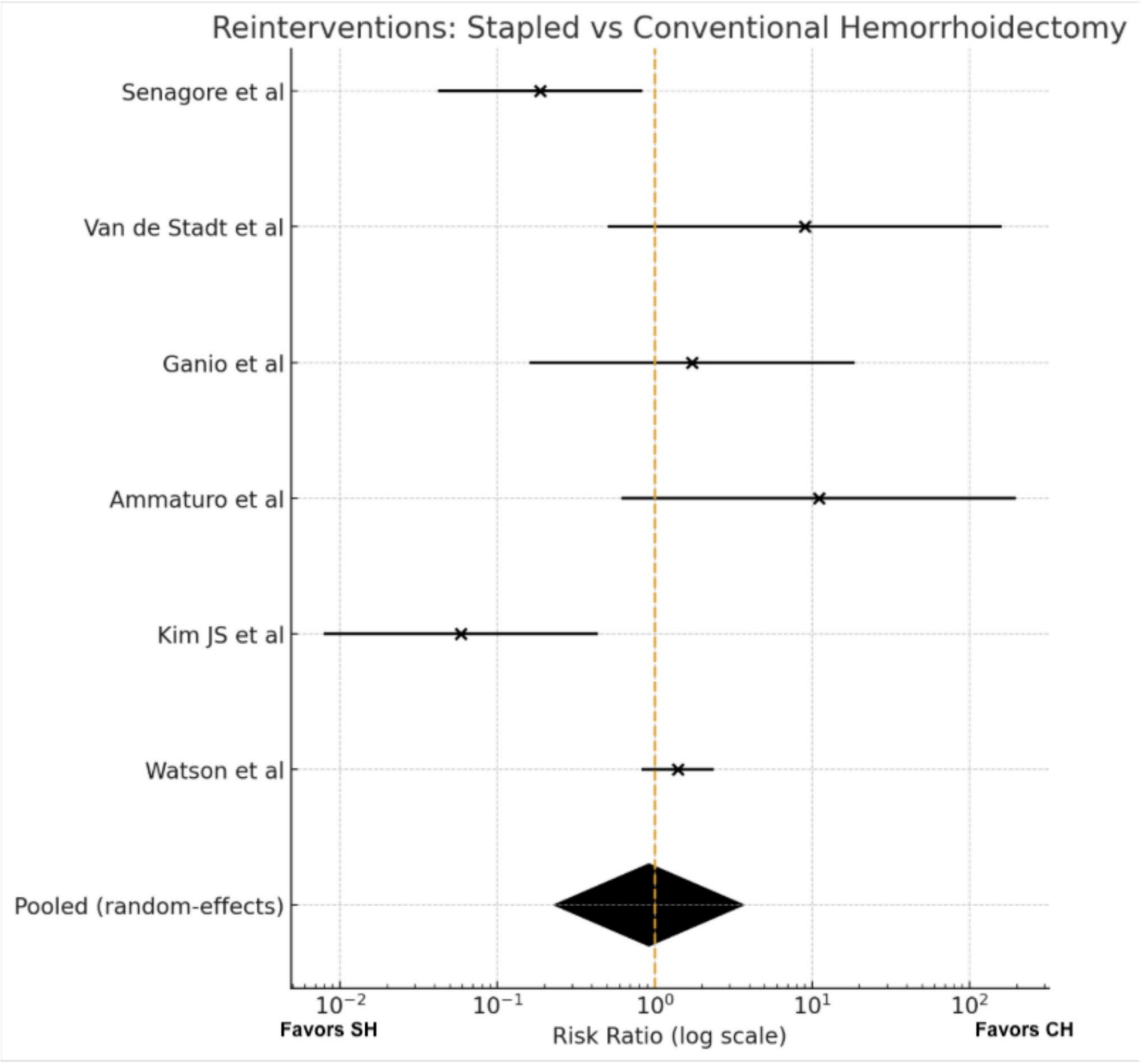
Additional operations

Six trials [11, 15, 18, 24–26] reported at least one postoperative reintervention and were therefore included in the quantitative synthesis. Ten additional studies with zero events in both treatment arms were non-informative for risk-ratio estimation and were excluded from the pooled analysis. Effect estimates varied substantially across the included trials, with some favouring SH and others favouring CH.

Using a random-effects model, no significant difference in reintervention rates was observed between stapled and conventional haemorrhoidectomy (RR 0.92, 95% CI 0.23–3.64; p = 0.90). Statistical heterogeneity was substantial (I^2^ = 74%), reflecting marked between-study variability and wide imprecision of the summary estimate. Sensitivity analyses based on alternative modelling assumptions produced consistent findings.

#### Anal pain

Postoperative anal or rectal pain was inconsistently reported across the included studies and occurred predominantly in the CH cohorts. Anal pain was documented in the CH arms of Ortiz et al. (2002) (n = 1) [21], Racalbuto et al. (n = 10) [23], Senagore et al. (n = 2) [18], Van de Stadt et al. (reported as pain during defecation, n = 3) [24], and Watson et al. (n = 10) [11]. Rectal pain was also noted in the CH group of Senagore et al. (n = 2) [18]. In the SH cohorts, postoperative pain was reported less frequently, with isolated cases described by Ortiz et al. (2002) (n = 1) [21], Van de Stadt et al. (pain during defecation, n = 6) [24], and Watson et al. (n = 6) [11]. Overall, postoperative pain appeared more common after CH, while SH cohorts reported fewer episodes and less consistent documentation.

#### Meta-analysis (Fig. 7)-Anal pain

**Fig. 7:**
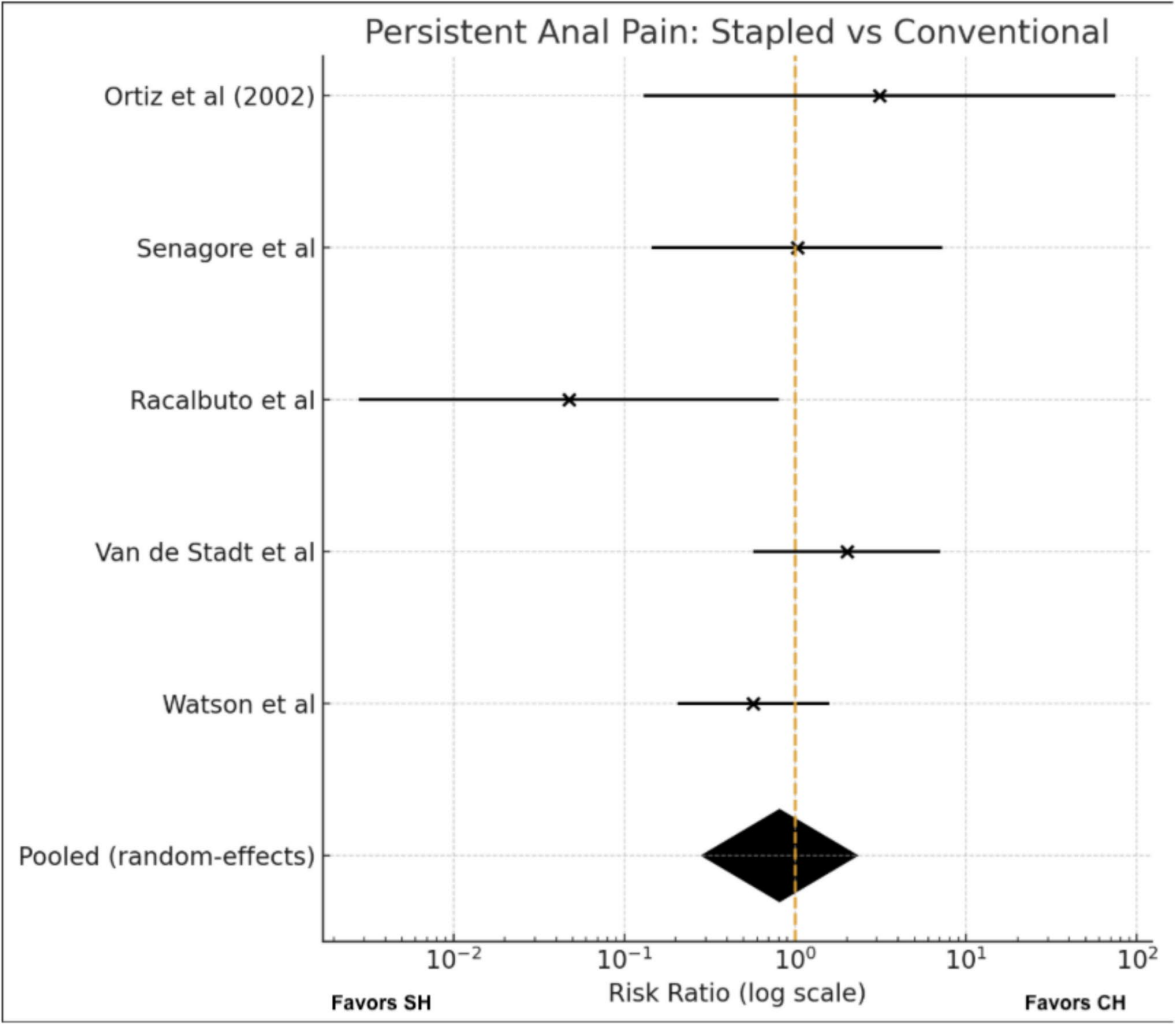
Persistent anal pain

Five trials [11, 18, 21, 23, 24] reported data on persistent anal pain and were included in the quantitative synthesis. Event rates were low and effect estimates varied considerably across studies, with some suggesting a higher risk after SH and others after CH.

Using a random-effects model, there was no significant difference in persistent anal pain between stapled and conventional haemorrhoidectomy (RR 0.81, 95% CI 0.29–2.29; p = 0.69). Heterogeneity was moderate (I^2^ = 44%), reflecting between-study variability and substantial imprecision.

#### Functional outcomes

Functional outcomes were variably reported across studies and included urgency, continence disturbances, soiling, sensory symptoms, incomplete evacuation, and urinary dysfunction.

Symptoms such as fecal urgency, soiling, incontinence to gas or liquids, and incomplete evacuation were described in both treatment groups, without a consistent pattern indicating superiority of one procedure. Urgency and sensory disturbances (itching, burning) appeared more frequently in SH cohorts in certain trials [15, 21, 28], whereas incomplete evacuation or difficult defecation occurred sporadically in both groups.

Transient incontinence to gas or liquids was reported in a limited number of studies [18, 26, 27] in both arms, and persistent incontinence was rare. Tenesmus, difficult defecation, and the sensation of incomplete evacuation were documented inconsistently across individual trials.

Urinary retention was observed in both SH and CH groups [11, 18], while dysuria was only occasionally reported.

#### Meta-analysis (Fig. 8)-Functional outcomes

**Fig. 8:**
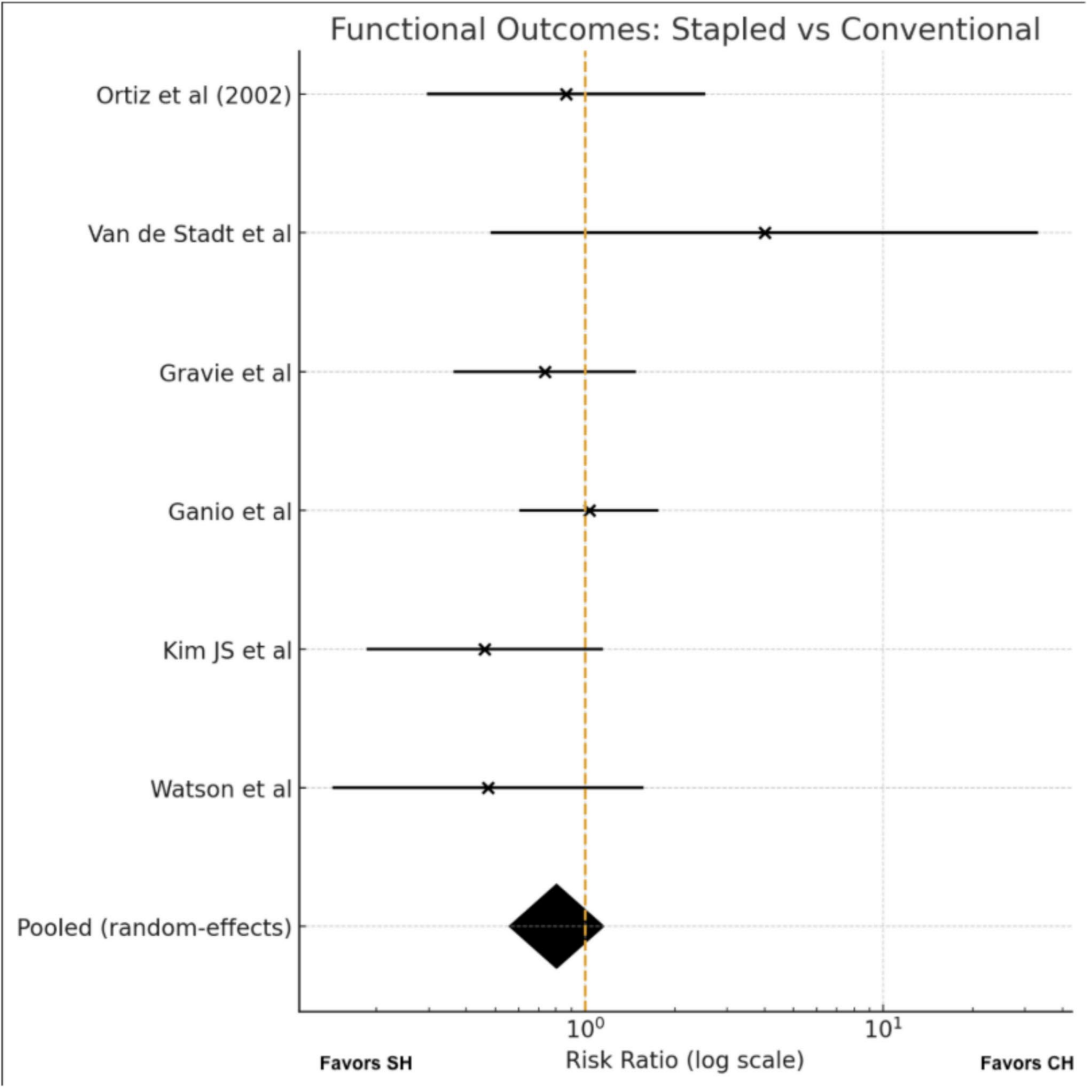
Functional outcomes

Six trials [11, 15, 21, 24, 26, 27] provided extractable data on postoperative functional impairment and were included in the quantitative synthesis. Event rates were generally low, and while the direction of effect varied minimally across studies, the confidence intervals were largely overlapping.

Using a random-effects model, no significant difference was found between stapled and conventional haemorrhoidectomy in terms of functional impairment (RR 0.80, 95% CI 0.56–1.15). Heterogeneity was low (I^2^ = 8%), indicating good consistency of effect estimates.

Overall, available evidence does not support a clinically meaningful advantage of either technique regarding postoperative functional morbidity.

#### Quality of life (QoL)

Quality-of-life outcomes were inconsistently reported and assessed using heterogeneous instruments, preventing formal meta-analysis. Only a minority of trials provided validated QoL measurements or long-term patient-reported outcomes.

Among studies assessing patient satisfaction, Ortiz et al. (2002) [21] found higher mean satisfaction in the CH group (9.3 vs 7.6 on a 0–10 scale). Smyth et al. [28] observed comparable satisfaction (CH 77% vs SH 80%). Gravié et al. [27], using a 0–10 Likert scale, reported similar QoL scores (CH 8.0 vs SH 7.8). Ammaturo et al. [25] described slightly higher satisfaction after CH (2.8 vs 2.06).

The eTHoS trial (Watson et al.) [11] reported a small but statistically significant advantage for CH using EQ-5D-3L AUC over 24 months (difference –0.073; p = 0.034). Nada et al. [19] reported Likert-based QoL results, although exact values were not disclosed.

Overall, the evidence suggests broadly comparable long-term QoL between SH and CH, with isolated studies showing marginal differences not consistently favouring one technique.

#### GRADE assessment (Suppl. Materials Table 2s)

According to the GRADE criteria, the certainty of evidence for overall recurrence was judged as low due to methodological limitations, moderate heterogeneity, and imprecision reinforced by the underpowered TSA. Prolapse-related recurrence reached a moderate level of certainty, as the effect was large and consistent despite some risk-of-bias concerns. Evidence for bleeding-related recurrence was low because of sparse events and wide confidence intervals. Reintervention showed very low certainty due to inconsistent reporting, marked heterogeneity, and severe imprecision. Persistent anal pain and functional outcomes similarly reached low certainty, largely because of heterogeneous definitions and limited event counts, while quality-of-life outcomes were rated very low due to substantial indirectness and inconsistent long-term assessment across trials.

## Discussion

The current management of haemorrhoidal disease increasingly relies on long-term, patient-centred outcomes rather than short-term postoperative advantages. Although SH was introduced as a less invasive alternative to CH, its true long-term effectiveness remains debated. In this context, our review provides the most up-to-date and comprehensive synthesis of randomized evidence with follow-up of at least twelve months, offering a clearer understanding of how these two procedures compare beyond the immediate postoperative period. Across 17 randomized trials [9, 11, 15–29] including more than 2,000 patients, we found that SH was associated with a significantly increased risk of long-term recurrence compared with CH (RR 1.56), although Trial Sequential Analysis indicated that the cumulative evidence remains underpowered. This finding should not be interpreted as contradictory, but rather as indicating a consistent signal of increased recurrence risk that has not yet reached the required information size to support definitive conclusions. The risk of recurrent prolapse was more than three times higher after SH (RR 3.28), with low heterogeneity, supporting the robustness of this finding. In contrast, long-term bleeding recurrence, reintervention rates, persistent anal pain, functional impairment, and QoL measures appeared broadly comparable between the two techniques, although reporting was often heterogeneous and based on different assessment tools. Collectively, these findings suggest that SH remains a safe and effective option with satisfactory long-term functional outcomes, although CH tends to provide more durable control of prolapse-related recurrence. Importantly, however, although overall recurrence rates appear numerically similar across procedures, the literature describes a subset of rare but potentially severe adverse events after SH—including pelvic sepsis, rectal perforation, staple-line dehiscence and rectovaginal fistula—that must be considered when selecting patients and counselling them preoperatively [30]. Conversely, complications more commonly associated with CH—such as anal stenosis —tend to be functionally relevant but are generally less immediately life-threatening. This qualitative difference highlights that similar overall complication rates may conceal clinically meaningful differences in severity and impact, reinforcing the importance of careful patient selection and informed consent. In advanced disease, particularly in patients with Grade IV haemorrhoids and predominant mucosal prolapse, CH remains the most established option in terms of long-term anatomical durability and is consistently supported by current guidelines [31–33]. However, SH may still represent a reasonable alternative in carefully selected patients who prioritise reduced postoperative pain and faster functional recovery, provided that the prolapse is mainly mucosal and that thorough preoperative counselling addresses the higher risk of prolapse-related recurrence observed in randomised evidence. In such scenarios, procedural choice should be individualised and guided by patient preferences, symptom burden, and an explicit discussion of the trade-off between early recovery and long-term durability.

Our results are broadly consistent with long-term concerns raised in previous studies [7, 34], which have similarly highlighted an increased risk of prolapse-related recurrence and a greater need for late symptom management after SH. Likewise, the earlier meta-analysis by Giordano et al. (2009) [10] confirmed this pattern, reporting a significantly higher risk of prolapse recurrence and a greater need for reintervention after SH, despite better short-term outcomes. By focusing exclusively on RCTs with ≥ 12 months of follow-up, our findings provide a contemporary, higher-level confirmation that the early postoperative advantages of SH do not translate into superior long-term durability.

Regarding functional outcomes, previous literature has suggested that SH may predispose to urgency, tenesmus or altered rectal sensitivity [27]. Although we observed a similar trend in descriptive data, our pooled analysis did not demonstrate statistically significant differences. These findings may be explained by variability in outcome reporting, non-standardised assessment methods, and the insufficient power of current studies to identify differences in low-frequency events.

Beyond these considerations, several limitations must also be acknowledged. Recurrence was not uniformly defined across trials, with prolapse-related and bleeding-related events variably reported, while QoL and functional outcomes were assessed using heterogeneous, often non-validated instruments, limiting comparability. Trial Sequential Analysis further showed that the accumulated sample size remains below the required information threshold, indicating that the current evidence is still underpowered and that additional high-quality trials are needed to confirm or refute clinically meaningful differences in long-term recurrence. Additional variability in CH technique—including open, closed, and energy-based approaches—introduces further heterogeneity that may influence outcomes. Nonetheless, this review has several strengths: it includes the largest and most contemporary pool of randomized trials with long-term follow-up, applies rigorous PRISMA methodology, uses predefined subgroup analyses to clarify which components of recurrence primarily contributed to the overall effect, and incorporates Trial Sequential Analysis to enhance the interpretability of the evidence base.

Collectively, these findings indicate that while SH remains a valid minimally invasive option for selected patients, CH appears to offer more durable long-term anatomical correction, particularly with respect to prolapse recurrence. Given the overall similarity in pain, functional outcomes, and QoL, procedural choice should be tailored to disease severity, symptom profile, and individual patient priorities within a shared decision-making framework.

## Supplementary Information

Below is the link to the electronic supplementary material.

Supplementary file1 (DOCX 269 KB)

Supplementary file2 (DOCX 557 KB)

## Data Availability

All data generated or analysed during this study are included in this published article and its supplementary materials. Additional datasets derived from the analyses are available from the corresponding author upon reasonable request.
