# Supplementary material for: Long-term outcomes of stapled haemorrhoidopexy versus conventional haemorrhoidectomy: An updated systematic review, meta-analysis and trial-sequential analysis of randomized controlled trials
Source: Int J Colorectal Dis. 2026 Jan 14;41(1):34. doi: 10.1007/s00384-026-05080-3 (PMC12808294; doi:10.1007/s00384-026-05080-3)
Supplement: Supplementary file 2 — Supplementary file2 (DOCX 557 KB) [file 384_2026_5080_MOESM2_ESM.docx]

# Risk of Bias 2 Summary

Legend:

🟢 Low risk – 🟡 Some concerns – 🔴 High risk

| Study (Year) | D1 | D2 | D3 | D4 | D5 | OVERALL |
| --- | --- | --- | --- | --- | --- | --- |
| Boccasanta 2001 | 🟡 | 🟢 | 🟡 | 🟡 | 🟡 | 🟡 |
| Shalaby 2001 | 🟡 | 🟢 | 🟢 | 🟡 | 🟡 | 🟡 |
| Ortiz 2002 | 🟡 | 🟢 | 🟡 | 🟡 | 🟡 | 🟡 |
| Hetzer 2002 | 🟡 | 🟢 | 🟢 | 🟡 | 🟡 | 🟡 |
| Smyth 2003 | 🔴 | 🟢 | 🟡 | 🟡 | 🟡 | 🔴 |
| Kairaluoma 2003 | 🟡 | 🟢 | 🟡 | 🟡 | 🟡 | 🟡 |
| Au-Yong 2003 | 🟡 | 🟢 | 🟡 | 🟡 | 🟡 | 🟡 |
| Racalbuto 2004 | 🟡 | 🟢 | 🟡 | 🟡 | 🟡 | 🟡 |
| Senagore 2004 | 🟡 | 🟢 | 🔴 | 🟡 | 🟡 | 🔴 |
| Gravié 2005 | 🟡 | 🟢 | 🟢 | 🟡 | 🟡 | 🟡 |
| Basdanis 2005 | 🟡 | 🟢 | 🔴 | 🟡 | 🟡 | 🔴 |
| Ortiz 2005 | 🟡 | 🟢 | 🟢 | 🟡 | 🟡 | 🟡 |
| Van de Stadt 2005 | 🟡 | 🟢 | 🟢 | 🟡 | 🟡 | 🟡 |
| Ganio 2007 | 🟡 | 🟢 | 🟡 | 🟡 | 🟡 | 🟡 |
| Ammaturo 2012 | 🟡 | 🟡 | 🟢 | 🟡 | 🟡 | 🟡 |
| Kim 2013 | 🟢 | 🟢 | 🟢 | 🟡 | 🟢 | 🟡 |
| Watson 2016 | 🟢 | 🟢 | 🔴 | 🟡 | 🟢 | 🔴 |
| Nada 2023 | 🟡 | 🟢 | 🟢 | 🟡 | 🟡 | 🟡 |

Publication bias analysis

1. Overall Recurrences


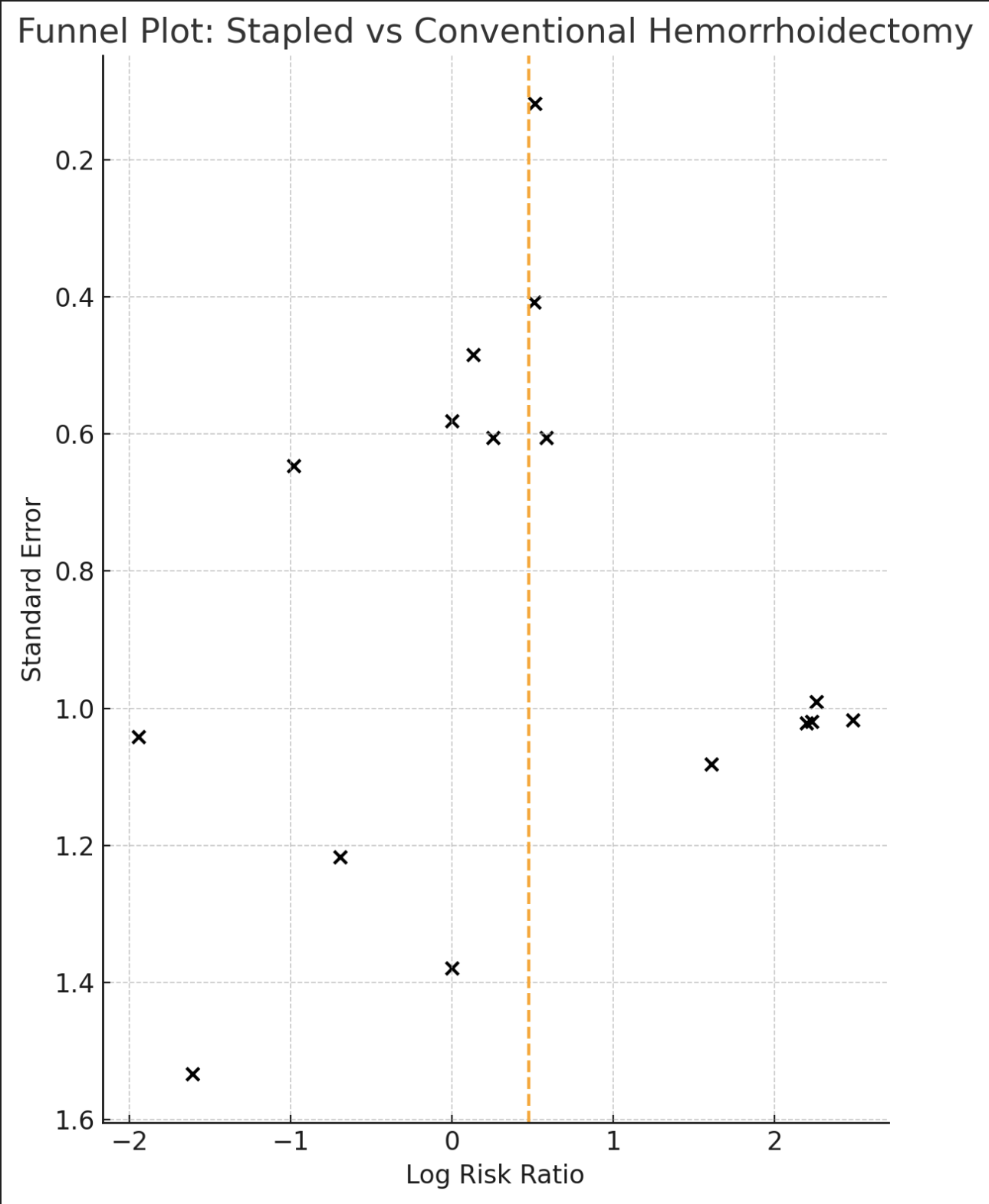


1. Bleeding recurrences


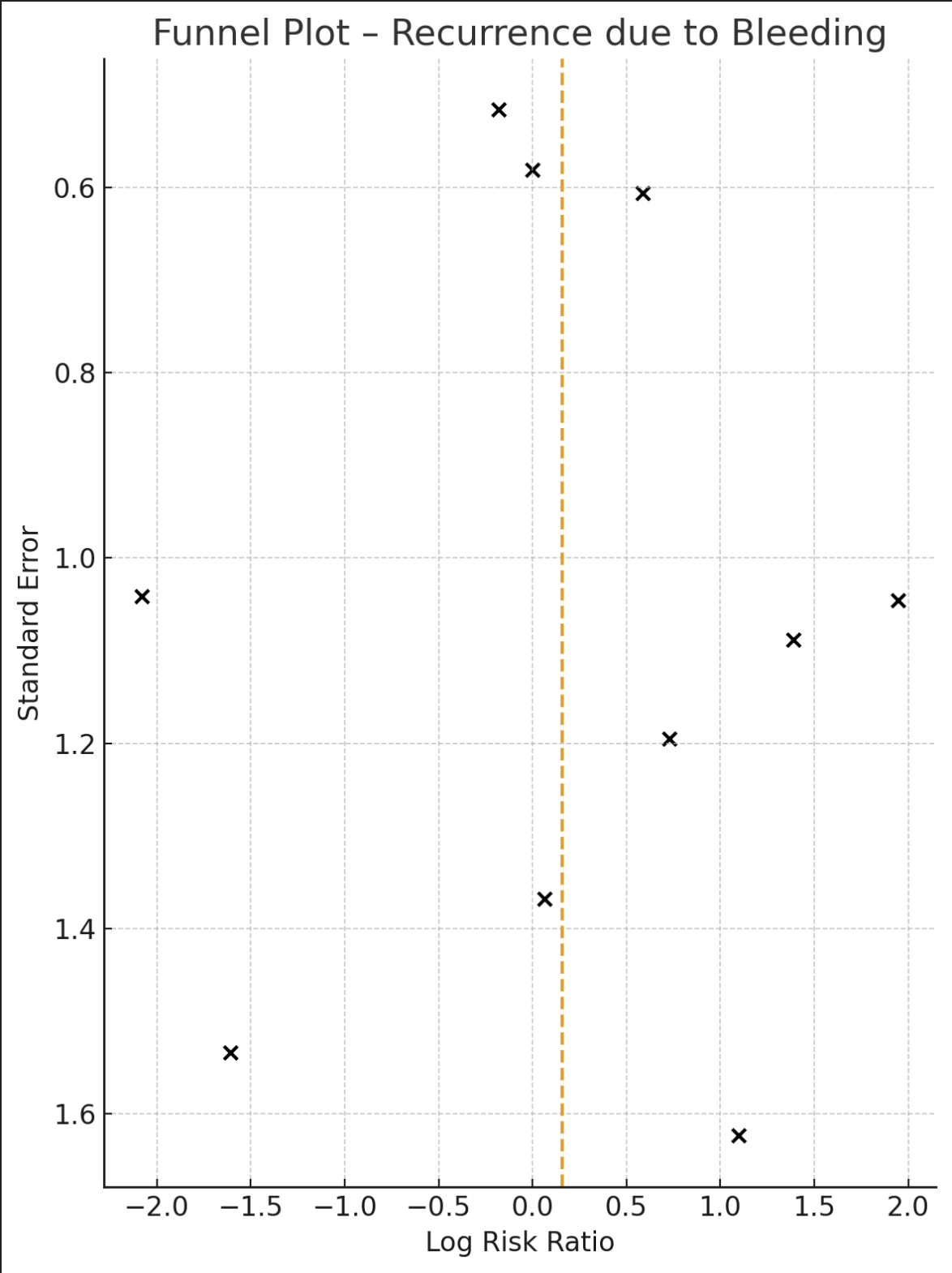


1. Prolapse recurrences


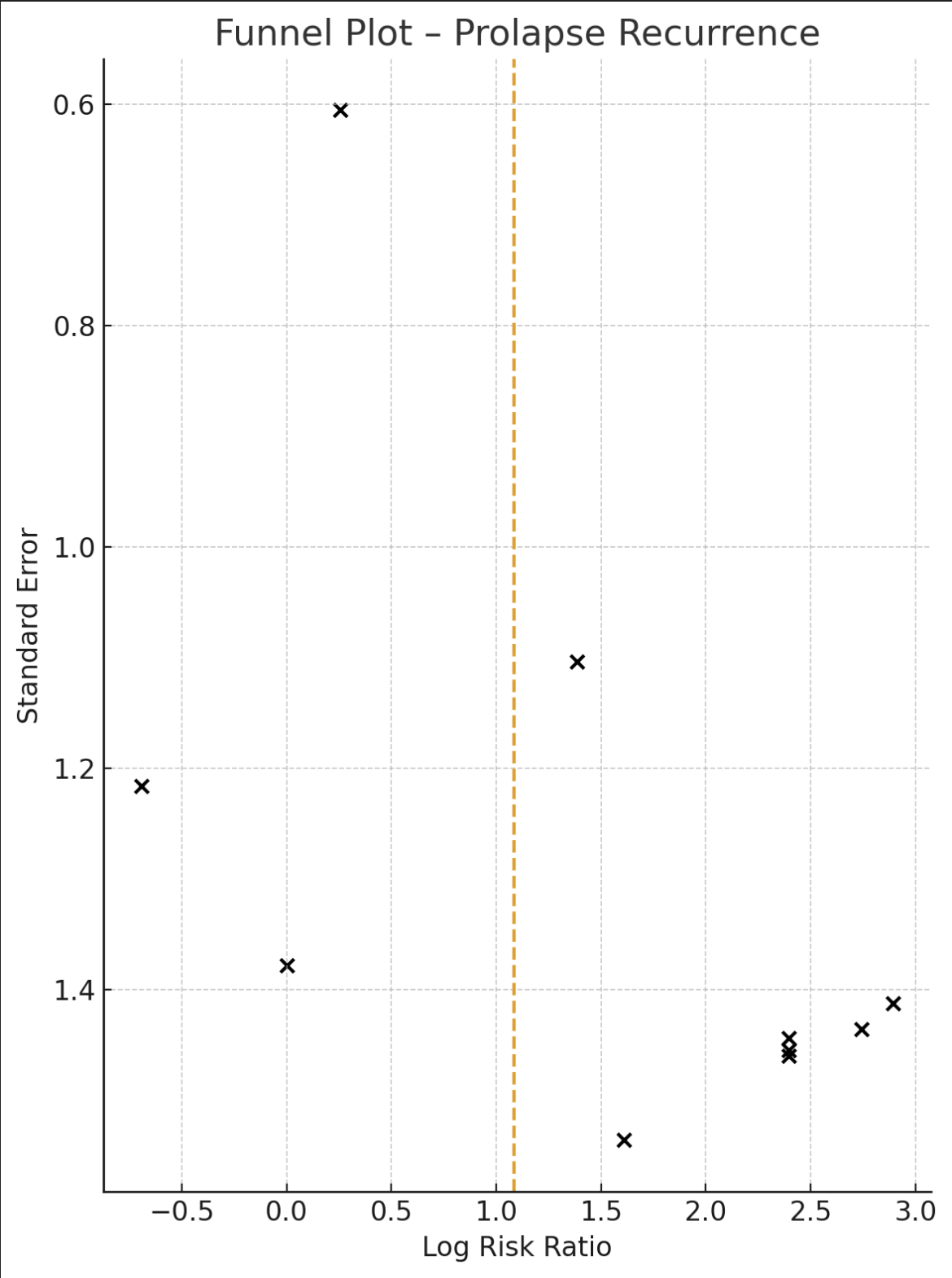


Sub-group analysis - Overall recurrences (Studies with follow up > 12 months)


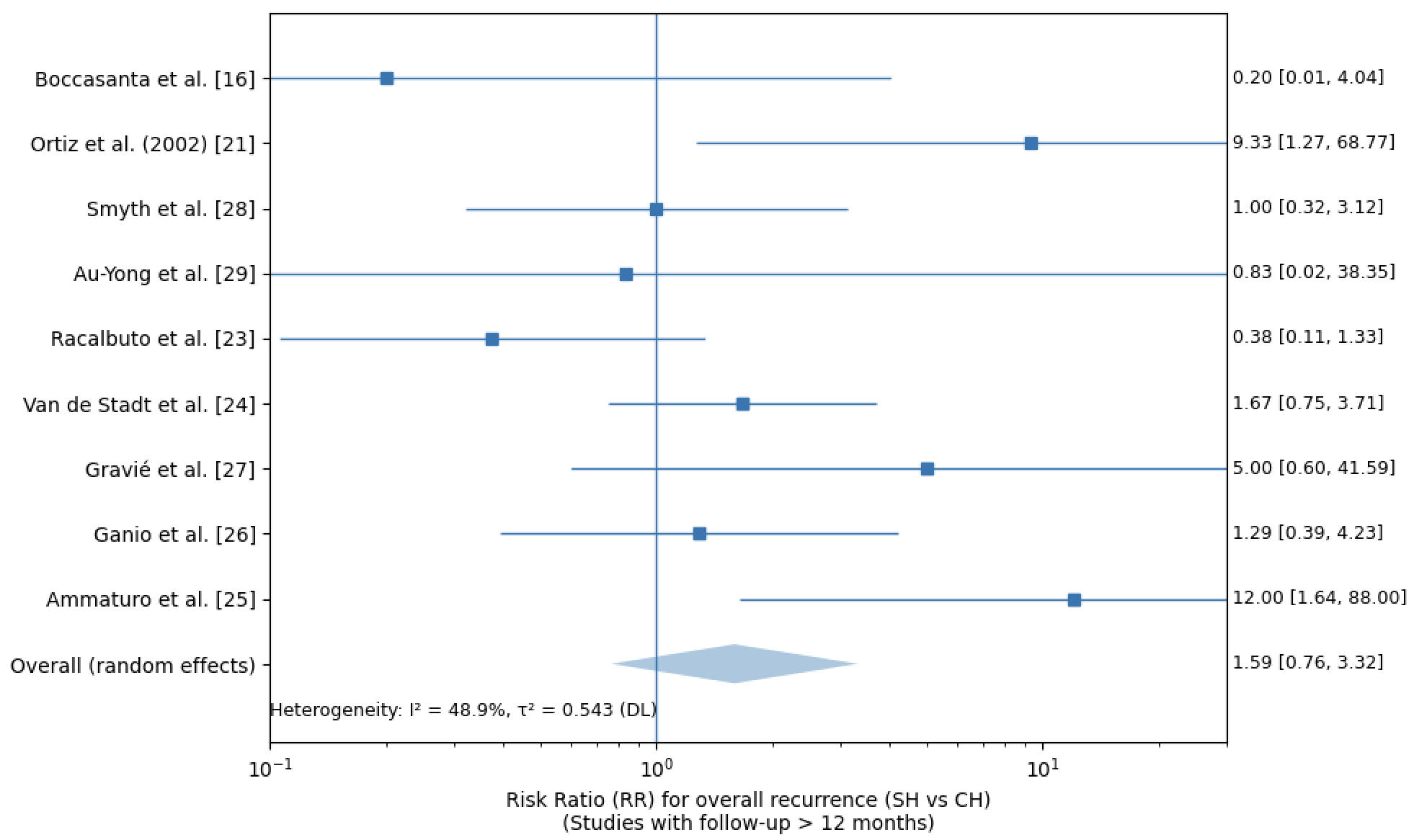


Forest plot of overall recurrence (prolapse and bleeding combined) comparing stapled hemorrhoidopexy (SH) versus conventional hemorrhoidectomy (CH), including only studies with a follow-up longer than 12 months. Pooled analysis using a random-effects model (DerSimonian–Laird) showed no significant difference between groups (RR 1.59, 95% CI 0.76–3.32; p = 0.21; I² = 48.9%).
